# Social determinants of health, clinical outcomes, and healthcare resource utilization in participants with and without narcolepsy in the *All of Us* research program

**DOI:** 10.1093/sleepadvances/zpag088

**Published:** 2026-08-05

**Authors:** Sarah C Markt, Caroleen Drachenberg, Anan Zhou, Katarina Irwin, Silky Beaty, Jon Michael Stroh, Martin Zagari, Jessica K Alexander, Marisa Whalen, Junko Saber, Barbara Hutchinson

**Affiliations:** Jazz Pharmaceuticals, Palo Alto, CA, United States; Jazz Pharmaceuticals, Palo Alto, CA, United States; USA Genesis Research Group, Hoboken, NJ, United States; Innopiphany, Irvine, CA, United States; Jazz Pharmaceuticals, Palo Alto, CA, United States; Innopiphany, Irvine, CA, United States; Innopiphany, Irvine, CA, United States; Jazz Pharmaceuticals, Palo Alto, CA, United States; Jazz Pharmaceuticals, Philadelphia, PA, United States; Innopiphany, Irvine, CA, United States; Chesapeake Cardiac Care, Bowie, MD, United States

**Keywords:** narcolepsy, cardiovascular medicine, coronary artery disease, epidemiology, healthcare utilization and costs, hypertension, medical economics, obesity, statistics

## Abstract

**Study Objectives:**

Evaluate the clinical and economic burden and impact of social determinants of health (SDoH) in narcolepsy.

**Materials and Methods:**

This retrospective, observational study used electronic health record and survey data from the *All of Us* Research Program (1/1/2009–7/31/2022). Participants with narcolepsy were matched 1:3 to participants without narcolepsy. Descriptive statistics and regression analyses (odds ratios [ORs]) summarized demographics, clinical comorbidities, and SDoH; hazard ratios (HRs) estimated risk of incident hypertension, hyperlipidemia, obesity, and coronary arteriosclerosis (CA); and incidence rate ratios (IRRs) estimated healthcare resource utilization (HCRU).

**Results:**

The sample comprised 2766 participants (narcolepsy, *n* = 694; non-narcolepsy, *n* = 2072; mean age, 49 years; 70 per cent female). Despite high education levels, greater proportions were out of work/the workforce in the narcolepsy versus non-narcolepsy cohort (38.5 per cent vs. 29.2 per cent). Among SDoH survey respondents, greater food insecurity, loneliness, and everyday/medical discrimination, and less social support/cohesion were reported in the narcolepsy (*n* = 259) versus non-narcolepsy (*n* = 768) cohort. Individuals with versus without narcolepsy had higher odds (OR [95 per cent confidence interval; CI]) of cardiovascular (1.76 [1.35–2.28]), cardiometabolic (2.12 [1.73–2.59]), and renal (2.47 [1.72–3.56]) comorbidities; higher risk (HR [95 per cent CI]) of developing hypertension (2.00 [1.51–2.65]), hyperlipidemia (1.96 [1.52–2.54]), obesity (2.42 [1.93–3.05]), and CA (1.75 [1.27–2.42]); and increased HCRU (IRR [95 per cent CI]) for inpatient days (2.07 [1.55–2.78]), outpatient visits (1.51 [1.35–1.70]), and emergency department visits (1.70 [1.19–2.43]).

**Conclusions:**

Narcolepsy is associated with employment barriers, SDoH-related disparities, and elevated comorbidity burden and risk. Comprehensive and timely management strategies are needed to address whole-person health.

## Graphical Abstract

**Graphical Abstract:**
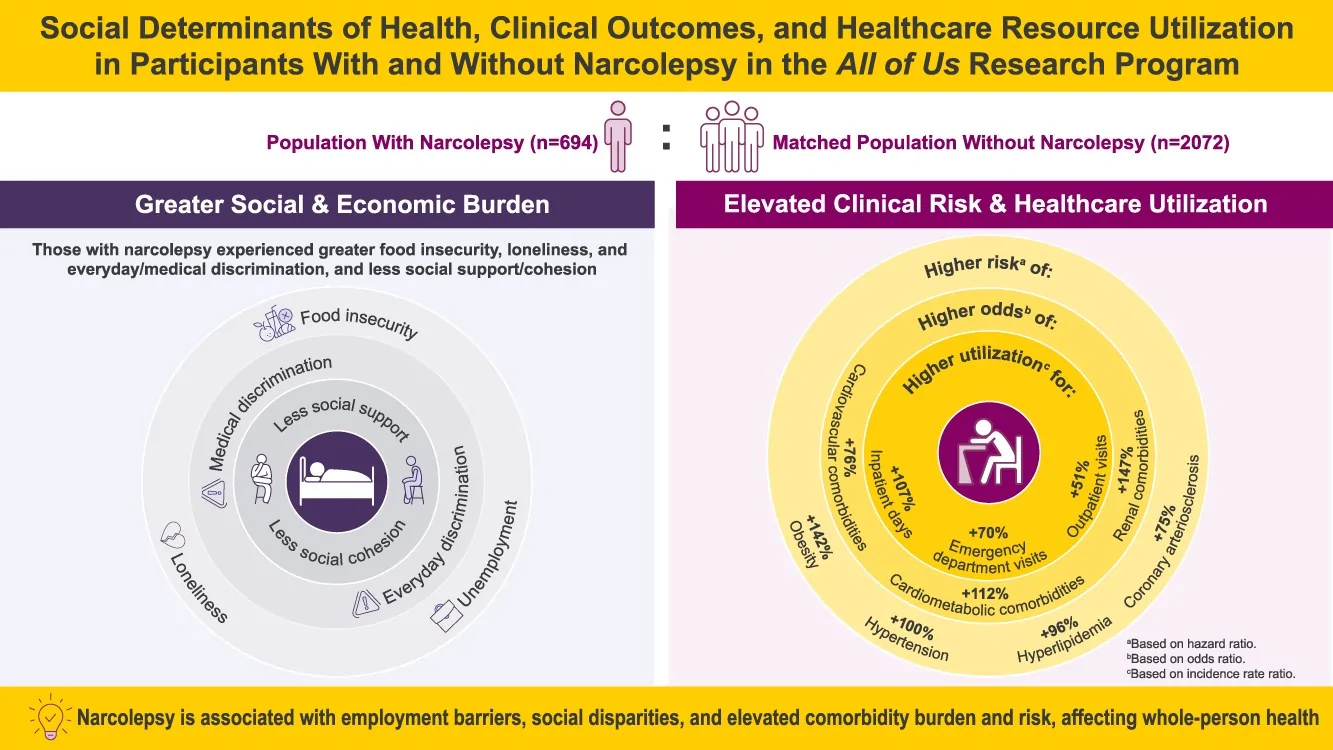

**Statement of Significance**

Narcolepsy impacts individuals across multiple domains, affecting economic (e.g. employment, disability), social determinants of health (SDoH; e.g. access to care, food insecurity), social (e.g. loneliness, support), and clinical (e.g. cardiovascular, cardiometabolic, cardiorenal, and psychiatric comorbidities) outcomes​. Economic and SDoH challenges compromise quality of life and may further amplify the prevalence and risk of comorbidities. Early identification and comprehensive management of narcolepsy are paramount to address whole-person health, including comorbidities and SDoH factors influencing care access, to reduce the clinical and economic burden people with narcolepsy experience, and to improve overall health outcomes.

## Introduction

Narcolepsy is a rare, chronic neurological disorder of hypersomnolence characterized by a pentad of symptoms, including excessive daytime sleepiness, cataplexy, disrupted nighttime sleep, sleep paralysis, and hypnagogic and/or hypnopompic hallucinations [1]. Narcolepsy is classified into two types: type 1 (NT1), in which cataplexy is present and/or cerebrospinal fluid (CSF) hypocretin-1 levels are low (≤110 pg/mL or less than one-third of mean values in healthy individuals), and type 2 (NT2), in which cataplexy is absent and CSF hypocretin-1 levels are higher than 110 pg/mL or more than one-third of mean values in healthy individuals [1].

Compared with individuals without narcolepsy, individuals with narcolepsy have a higher prevalence and risk of developing several comorbid conditions, including cardiovascular (CV), cardiometabolic (CM), and cardiorenal (CR) diseases, as well as neurological and psychiatric comorbidities, including depression, anxiety, and bipolar disorder [2–8]. Individuals with narcolepsy also have a higher risk of conditions that often lead to CV disease, including hypertension, diabetes, and hyperlipidemia [3, 5, 6], even early in life, with heightened risk observed in individuals younger than 25 years of age [9]. Compared with individuals without narcolepsy, individuals with narcolepsy have an increased risk of developing stroke, heart failure, ischemic stroke, major adverse cardiac events, atrial fibrillation, edema, and CV disease [5, 9]. Additionally, chronic use of certain treatments for narcolepsy (such as stimulants and high-sodium oxybates) may further exacerbate these CV risks [10–12].

Individuals with narcolepsy may experience substantially higher healthcare resource utilization (HCRU), including increased rates of inpatient admissions, emergency department (ED) visits, and consultations with neurologists and psychiatrists [2]. The annual direct medical costs for individuals with narcolepsy are nearly double those of matched controls without narcolepsy ($11 702 vs. $5261), underscoring the financial burden on both patients and healthcare systems [13, 14]. The symptoms and costs of narcolepsy severely impact health-related quality of life, with studies documenting social stigma, the unpredictability of symptoms, and patient dissatisfaction as primary concerns affecting individuals with narcolepsy [2, 13]. Despite these frequent and persistent symptoms, diagnosis may be delayed by upwards of 15 years [15, 16].

While the clinical and economic burden of narcolepsy is well-documented, the role of social determinants of health (SDoH), such as discrimination, income, and food insecurity, in narcolepsy has not generally been addressed; nor has research explored how SDoH factors may influence CV, CM, and CR comorbidities or the risk of CV/CM/CR events in this group. The absence of robust SDoH data in existing studies hinders the ability to identify vulnerable subgroups who may face additional barriers to care, such as delayed diagnosis and limited access to treatments. Launched by the National Institutes of Health in May 2018, the *All of Us* Research Program is a nationwide initiative created to improve healthcare through research that includes participants who reflect the diversity of the US population. In particular, underrepresented groups, including racial and ethnic minorities, are intentionally oversampled, and therefore, the population may not be nationally representative.

*All of Us* integrates electronic health records (EHR) and survey data incorporating lifestyle, environmental, genetic, and SDoH factors. Much of the individual-level participant data is self-reported, collected cross-sectionally through surveys. All participants complete three core surveys (“The Basics,” “Lifestyle,” and “Overall Health”), while additional elective surveys completed by some participants assess information on SDoH factors (“Social Factors of Health”) and access to care (“Health Care Access and Utilization”). Data are anonymized, with the additional safeguard of censoring data for any groups of fewer than 20 participants; *All of Us* complies with the Health Insurance Portability and Accountability Act. To date, more than 849 000 participants have enrolled, and as of July 1, 2022, a total of 413 457 participants completed surveys.

Using survey data from the *All of Us* Research Program, the current study aimed to (1) characterize and describe the prevalence of comorbid conditions and HCRU outcomes in participants with narcolepsy compared with participants without narcolepsy, overall and stratified by household income, and (2) estimate the risk of developing CV/CM/CR-related conditions among participants with narcolepsy compared with participants without narcolepsy.

## Materials and methods

### Study design and participant population

This was a retrospective, observational study that included participants from the *All of Us* Research Program.

**Figure 1:**
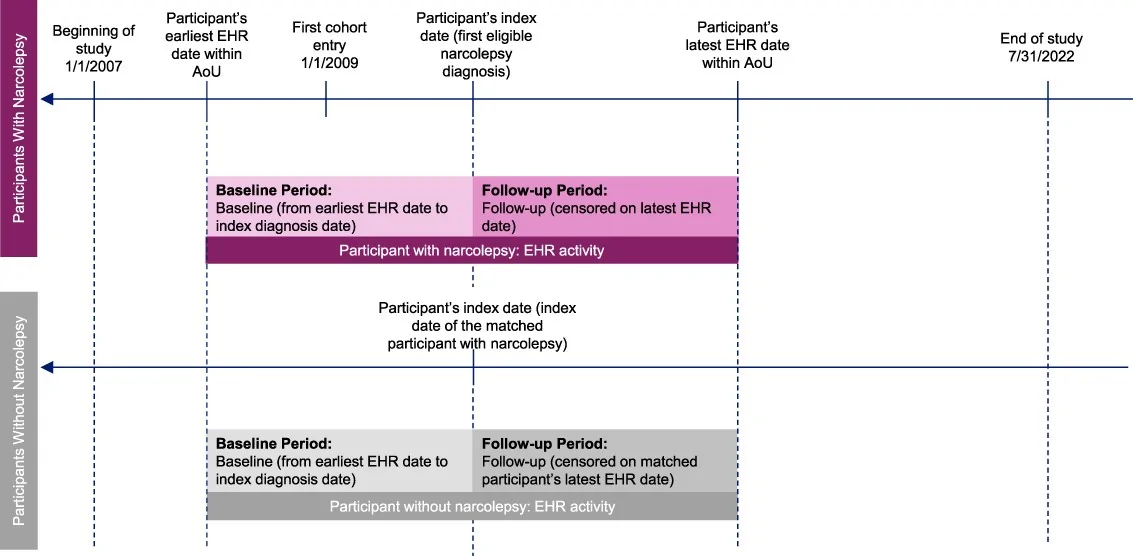
Study design. AoU, *All Of Us*; EHR, electronic health record.

Data were available from July 1, 2007, to July 31, 2022, with the participant identification period occurring between January 1, 2009, and January 31, 2022 (Figure 1). This time frame was selected based on improvements in data quality, consistency, and comprehensiveness of EHRs following the implementation of the Health Information Technology for Economic and Clinical Health Act of 2009 [17].

Participants with narcolepsy were identified and included based on having at least one Systematized Nomenclature of Medicine–Clinical Terms (SNOMED CT) code for a confirmed narcolepsy diagnosis (60380001, 193042000), consented to EHR record transmission, completed the core surveys, and had a baseline and follow-up duration of ≥1 day. Eligible individuals without narcolepsy included those who consented to EHR record transmission, completed the core surveys, did not have a SNOMED CT code present for narcolepsy or idiopathic hypersomnia (3731000119107), and answered “no” to the question, “Including yourself, who in your family has had narcolepsy?” in the “Personal and Family Health History” survey. Logistic regression was used to calculate propensity scores, and individuals with narcolepsy were propensity score–matched 1:3 to individuals without narcolepsy based on sex at birth, age at the time of The Basics survey completion, completion of the SDoH survey (yes/no), earliest recorded EHR date, and time in the EHR system (number of days from the earliest record in the study period to the final record in the study period or the end of the study period, whichever was sooner). Exact matching constraints were applied for SDoH survey completion status and earliest EHR date to maintain precision in these key demographic and temporal factors. The matching process was completed in a single integrated step by using the propensity scores, nearest-neighbor matching was performed within each stratum of participants with and without narcolepsy sharing identical values for exact-matched variables. A small number of participants did not have a perfect 1:3 match due to the absence of available individuals who met the criteria for matching. Specifically, the non-narcolepsy cohort is nine participants smaller than expected for this reason. One participant without narcolepsy was dropped due to negative follow-up time.

The index date for the narcolepsy cohort was defined as the earliest recorded diagnosis of narcolepsy within the identification period. For the non-narcolepsy cohort, the index date was matched to the corresponding narcolepsy case diagnosis date. The baseline period was defined as the time from a participant’s earliest recorded EHR entry after the study start date up to their index date (Figure 1). The follow-up period extended from the index date until the earliest occurrence of death, loss of clinical activity (defined as the final recorded EHR entry before the study end), or the end of the study period (July 31, 2022). For the non-narcolepsy cohort, follow-up continued until the earliest of death, loss of clinical activity for their matched case, or the study end date.

### Demographics and SDoH variables from surveys

Demographics and SDoH were identified from participant surveys, including The Basics, Overall Health, Health Care Access and Utilization, and Social Factors of Health surveys. From the core surveys (The Basics, Overall Health, and Lifestyle), information included age, sex at birth, race/ethnicity, country of origin, Area Deprivation Index, education level, income, employment status, health insurance, and disability status. Variables obtained from the Health Care Access and Utilization survey included usual place for care, last seen provider, provider type seen in the last 12 months, needed care and could not afford, and delayed care for any reason. Additionally, the SDoH-specific survey, completed by a subset of the population, included metrics of neighborhood physical disorder, neighborhood social disorder, food insecurity, loneliness, everyday discrimination, medical discrimination, social support, social cohesion, and perceived stress. These scales were validated and scored according to Tesfaye *et al*. [18] who outlined the original scoring methods and transformations applied to each scale for *All of Us*.

### Clinical characteristics and HCRU from EHR

Clinical characteristics, including CV, CM, CR, psychiatric, and neurological conditions, were identified in the EHR using SNOMED CT codes. In addition to being defined by SNOMED CT code, hypertension was also defined by medication use, including angiotensin-converting enzyme inhibitors, alpha-2 agonists, angiotensin II receptor blockers, beta blockers, calcium channel blockers, potassium-sparing diuretics, renin inhibitors, thiazide diuretics, and vasodilators. Additionally, composite comorbidity outcomes were assessed, comprising any CV disease; any CM disease; any CR disease; and any CV, CM, or CR disease. Any CV comorbidity included angina pectoris, atrial fibrillation, heart failure, myocardial infarction, cardiac arrest, coronary arteriosclerosis, hypertension, and stroke. Any CM comorbidity included hyperlipidemia, type 2 diabetes, obesity, and metabolic encephalopathy. Any CR comorbidity included chronic kidney disease and hypertensive renal disease. The specific codes used for each condition are provided in Table S1.

The prevalence of each condition was assessed in the baseline period in both the narcolepsy and non-narcolepsy cohorts. The incidence of hypertension, hyperlipidemia, obesity, and coronary arteriosclerosis was assessed in the follow-up period, among those without prevalent disease for each respective outcome. HCRU was also derived from the EHR data, and included outpatient, inpatient, and ED visits.

### Statistical analysis

Descriptive summary statistics were calculated for all demographic, SDoH, and clinical characteristics, including means and standard deviations for continuous variables and frequencies and percentages for binary or categorical variables.

Logistic regression was used to calculate odds ratios (ORs) and 95 per cent confidence intervals (CIs) for the association between narcolepsy and baseline CV/CM/CR comorbidities, including hypertension, hyperlipidemia, obesity, any CV, any CM, and CR, and any CV/CM/CR comorbidities. Models were adjusted for age at index, sex at birth (male, female, unknown), time in the EHR, race/ethnicity (non-Hispanic White, non-Hispanic Black, Hispanic, non-Hispanic Asian/Pacific Islander, other, unknown), annual income (<$50 k, ≥$50 k), education (college graduate, some college, high school/GED, no high school, unknown), employment status (employed, retired, out of work, not in workforce, unknown), disability status (yes, no, missing), and insurance type (commercial, Medicare, Medicaid, uninsured, other, prefer not to answer). Obesity was additionally adjusted for in all models in which obesity was not the outcome of interest or included in the outcome of interest (e.g. CM). Furthermore, to examine whether the association of narcolepsy with baseline comorbidities varied by income, logistic regression models were used, stratifying by annual income level (lower income: <$50 k and higher income: ≥$50 k). To statistically compare estimates across strata, Wald tests (*P* interaction) were used.

The incidence of hypertension, hyperlipidemia, obesity, and coronary arteriosclerosis was assessed in the follow-up period. Unadjusted incidence rates with 95 per cent CIs were calculated per 100 person-years as the number of events divided by the person-time in years multiplied by 100 for total follow-up, as well as at 1, 3, and 5 years of follow-up. Participants must have had a minimum of 1, 3, and 5 years of follow-up to contribute to those respective calculations. Cox proportional hazards models were used to calculate adjusted hazard ratios (HRs) and 95 per cent CIs for total follow-up, and at 1, 3, and 5 years of follow-up. Models were adjusted for the same covariates as in the logistic regression models above. The proportional hazards assumption was checked with Schoenfeld residuals; to address violations of the assumption, right censoring was implemented across the 1-, 3-, and 5-year follow-up windows.

For HCRU outcomes, Tweedie generalized linear models were used due to the large number of zeros present in these measures (i.e. many participants had no inpatient, outpatient, or ED visits in baseline or follow-up) and were modeled per person per month (PPPM). Models were adjusted for age at diagnosis, race/ethnicity, income, education, employment status, insurance type, Charlson Comorbidity Index, 2017 Area Deprivation Index, disability status, regular place for healthcare advice, and delayed care.

Analyses were conducted in R versions 4.4.2, 4.4.3, and 4.5.0. Matching was performed using the *MatchIt* package in R.

## Results

### Participant demographic characteristics

A total of 413 360 *All of Us* participants completed surveys, and 286 856 had survey and EHR data. Of these, 694 participants had a diagnosis of narcolepsy and were matched to 2072 participants without narcolepsy. The median duration of the baseline and follow-up periods was 5.1 and 4.5 years, respectively. While participants with narcolepsy were more frequently non-Hispanic White compared with participants without narcolepsy, the narcolepsy cohort in this study was more ethnically diverse (11.8 per cent non-Hispanic Black and 8.4 per cent Hispanic) than in prior narcolepsy studies [4, 19] (Table 1). The narcolepsy cohort reported higher education levels than the non-narcolepsy cohort (at least some college education: 80.0 per cent vs. 69.7 per cent). However, the narcolepsy cohort was more likely than the non-narcolepsy cohort to be out of work or not in the workforce (38.5 per cent vs. 29.2 per cent) and slightly more likely to report an annual income less than $25 000 (27.4 per cent vs. 23.1 per cent). Participants with narcolepsy were also more likely to be on Medicare (32.6 per cent vs. 23.2 per cent).

**Table 1:** Baseline characteristics among participants with narcolepsy and matched participants without narcolepsy

	Narcolepsy cohort *n* = 694	Non-Narcolepsy cohort *n* = 2072
**Age at survey completion, years, mean (SD)**	48.0 (16.8)	49.2 (16.4)
**Age at diagnosis, years, mean (SD)**	45.5 (16.3)	49.2 (16.4)
**Sex, *n* (%)**		
Female	496 (71.5)	1449 (69.9)
**Race/ethnicity, *n* (%)**		
Non-Hispanic Asian/NHPI	22 (3.2)	47 (2.3)
Non-Hispanic Black	82 (11.8)	383 (18.5)
Hispanic	58 (8.4)	391 (18.9)
Non-Hispanic White	466 (67.1)	1115 (53.8)
Other	36 (5.2)	77 (3.7)
Missing/no answer	30 (4.3)	59 (2.8)
**Country of origin, *n* (%)**		
USA	637 (91.8)	1738 (83.9)
**2017 Area Deprivation Index, *n* (%)**	694	2071
Tertile 1	237 (34.1)	726 (35.1)
Tertile 2	275 (39.6)	732 (35.3)
Tertile 3	182 (26.2)	613 (29.6)
**Education level, *n* (%)**		
No high school diploma	25 (3.6)	170 (8.2)
High school diploma/GED	94 (13.5)	389 (18.8)
Some college	226 (32.6)	536 (25.9)
College graduate	329 (47.4)	909 (43.9)
Missing/no answer	20 (2.9)	68 (3.3)
**Annual income, *n* (%)**		
Less than $25 000	190 (27.4)	479 (23.1)
$25 000–$49 999	134 (19.3)	354 (17.1)
$50 000–$99 999	138 (19.9)	387 (18.7)
$100 000+	118 (17.0)	444 (21.4)
Missing/no answer	114 (16.4)	408 (19.7)
**Employment status, *n* (%)**		
Employed	270 (38.9)	1020 (49.2)
Not in workforce	206 (29.7)	430 (20.8)
Out of work	61 (8.8)	174 (8.4)
Retired	131 (18.9)	367 (17.7)
Missing/no answer	26 (3.7)	81 (3.9)
**Health insurance, *n* (%)**		
Private	260 (37.5)	872 (42.1)
Medicare	226 (32.6)	480 (23.2)
Medicaid	126 (18.2)	435 (21.0)
Other	54 (7.8)	122 (5.9)
Missing/no answer	27 (3.9)	152 (7.3)
**Medication exposure,** * ***n* (%)**		
Treatments used in narcolepsy	383 (55.2)	99 (4.8)
Oxybates^†^	49 (7.1)	0 (0.0)
Wake-promoting agents^‡^	283 (40.8)	21 (1.0)
Stimulants^‡^	230 (33.1)	89 (4.3)
Antidepressants	487 (70.2)	760 (36.7)
Antihypertensives	491 (70.7)	1056 (51.0)
Lipid-modifying agents	387 (55.8)	902 (43.5)
Treatments used in diabetes	278 (40.1)	585 (28.2)

^*^Occurring during the study period of July 1, 2007, to July 31, 2022. Medication exposure categories were not mutually exclusive; patients may have received multiple medications and could be counted in more than one category.

^†^Oxybate treatments include low-sodium oxybate, high-sodium oxybate and authorized generics, and fixed-dose high-sodium oxybate.

^‡^Wake-promoting agents include modafinil and armodafinil. Stimulant treatments include methylphenidate and amphetamines. Because medication indications were not available, stimulant and wake-promoting agent use may represent treatment for conditions other than narcolepsy.

GED, General Educational Development; NHPI, Native Hawaiian and Pacific Islander; USA, United States of America; SD, standard deviation.

**Figure 2:**
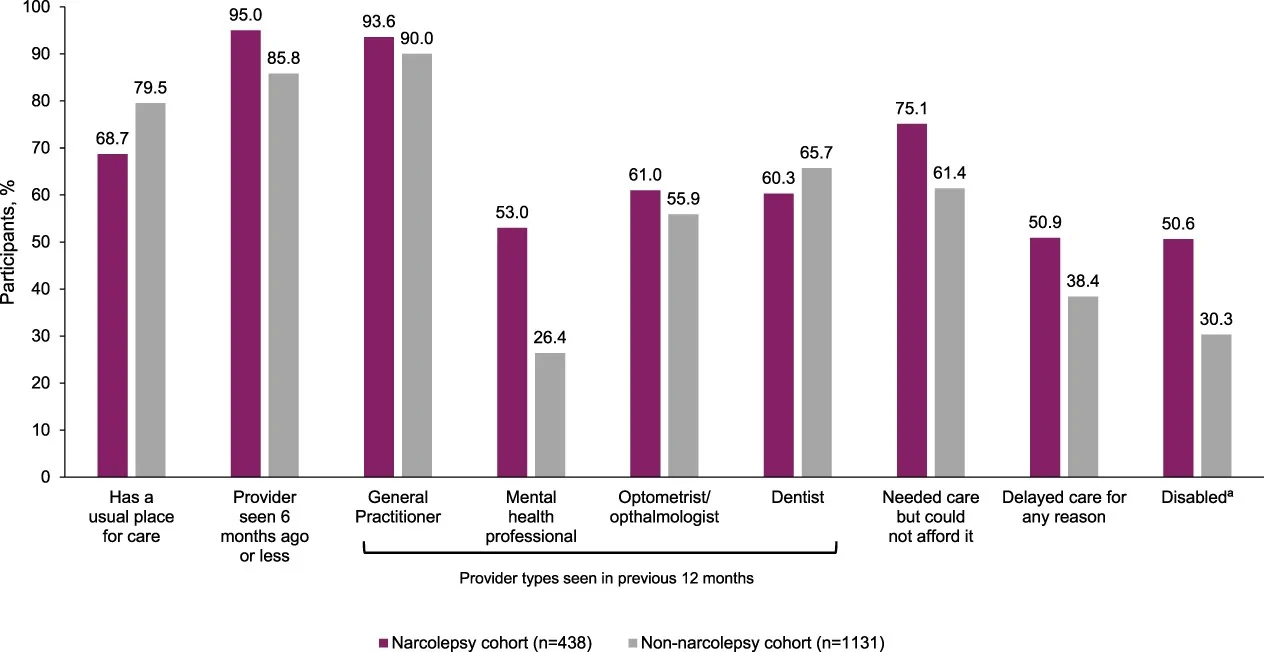
Healthcare access and utilization among participants with narcolepsy and matched participants without narcolepsy who completed the health care access and utilization survey. ^a^Disability status was reported in The Basics survey (narcolepsy cohort: *n* = 253; non-narcolepsy cohort: *n* = 709).

Among the participants who completed the Health Care Access and Utilization survey, participants with narcolepsy were more likely than those without narcolepsy to have seen a healthcare provider in the last 6 months (95.0 per cent vs. 85.8 per cent) and to have seen a general practitioner (93.6 per cent vs. 90.0 per cent) or mental health professional in the last 12 months (53.0 per cent vs. 26.4 per cent) (Figure 2). Participants with narcolepsy were also more likely to report that they needed care and could not afford it (75.1 per cent vs. 61.4 per cent) or had to delay care for any reason (50.9 per cent vs. 38.4 per cent) compared with participants without narcolepsy. In addition, disability, reported on The Basics survey, was more frequent in the narcolepsy cohort versus the non-narcolepsy cohort (50.6 per cent vs. 30.3 per cent).

**Figure 3:**
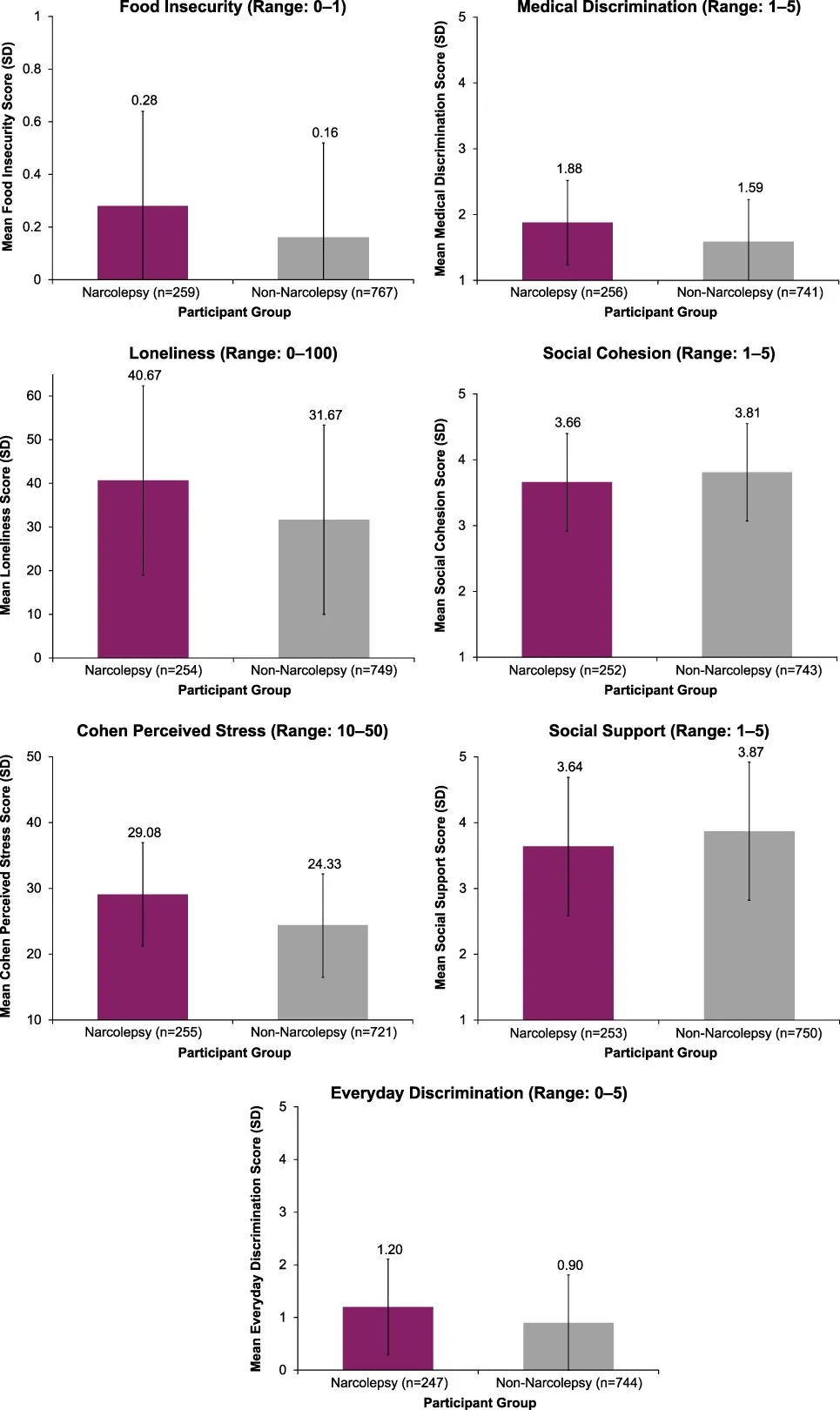
SDoH factor scales in participants with and without narcolepsy who completed the SDoH survey^a^. ^a^Higher scores indicate worse outcomes for all factors except Social Support and Social Cohesion. SD, standard deviation; SDoH, social determinants of health.

Among those who completed the SDoH survey (Figure 3), participants with narcolepsy reported higher levels of loneliness (40.7 vs. 31.7, from a total possible range of 0–100) and food insecurity (0.28 vs. 0.16, from a total possible range of 0–1), and marginally higher levels of perceived stress (29.1 vs. 24.3; total possible range of 10–50), everyday discrimination (1.20 vs. 0.90; total possible range of 0–5), and medical discrimination (1.88 vs. 1.59; total possible range of 1–5), compared with participants without narcolepsy.

### Comorbidity prevalence

**Figure 4:**
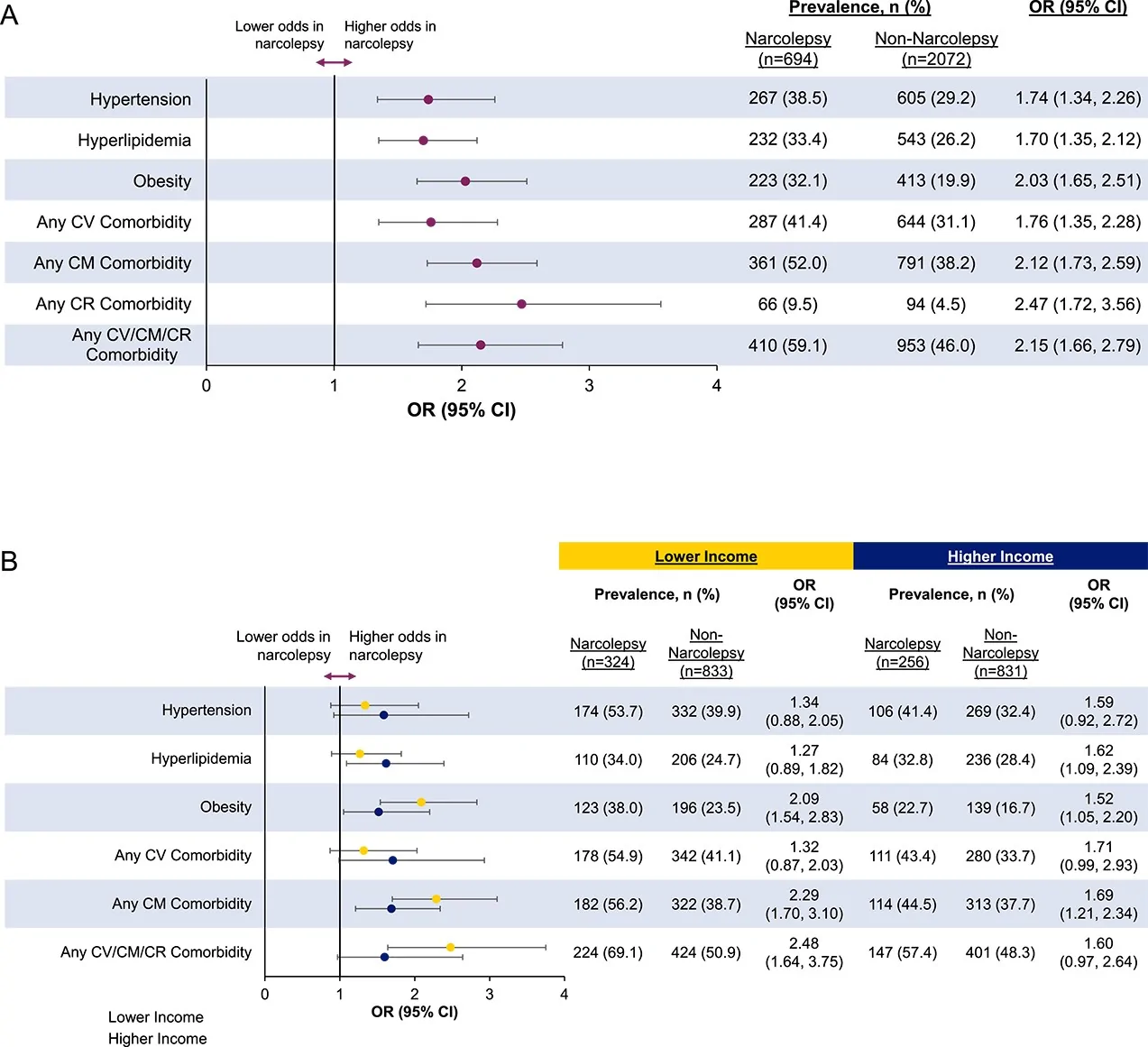
Association between narcolepsy and odds of prevalent cardiovascular,^a^ cardiometabolic,^b^ or cardiorenal^c^ comorbidities overall (A) and stratified by income (B). ^a^CV comorbidities were angina pectoris, atrial fibrillation, heart failure, myocardial infarction, cardiac arrest, coronary arteriosclerosis, hypertension, and stroke. ^b^CM comorbidities were hyperlipidemia, type 2 diabetes mellitus, obesity, and metabolic encephalopathy. ^c^CR comorbidities were chronic kidney disease and hypertensive renal disease. The composite outcome for any CR comorbidity is not shown for analyses stratified by income in order to comply with the *All of Us* data dissemination policy (data censored for outcomes reported for <20 participants). CI, confidence interval; CM, cardiometabolic; CR, cardiorenal; CV, cardiovascular; OR, odds ratio.

The narcolepsy cohort had a higher prevalence of CV, CM, and CR comorbidities at baseline, compared with the non-narcolepsy cohort, including hypertension (48.7 per cent vs. 36.3 per cent), hyperlipidemia (33.4 per cent vs. 26.2 per cent), and obesity (32.1 per cent vs. 19.9 per cent) (Table 2). Psychiatric and neurological disorders were also more prevalent at baseline in the narcolepsy cohort compared with the non-narcolepsy cohort, including depressive disorder (45.7 per cent vs. 20.1 per cent) and musculoskeletal pain (50.0 per cent vs. 31.9 per cent) (Table 2). In adjusted models, the narcolepsy cohort had higher odds of having obesity (OR: 2.03, 95 per cent CI: 1.65, 2.51), hypertension (OR: 1.74, 95 per cent CI: 1.34, 2.26), and hyperlipidemia (OR: 1.70, 95 per cent CI: 1.35, 2.12). Similar results were observed for the composite outcomes, such that the narcolepsy cohort had higher odds of any CV (OR: 1.76, 95 per cent CI: 1.35, 2.28), any CM (OR: 2.12, 95 per cent CI: 1.73, 2.59), any CR (OR: 2.47, 95 per cent CI: 1.72, 3.56), and any CV/CM/CR (OR: 2.15, 95 per cent CI: 1.66, 2.79), compared with the non-narcolepsy cohort (Figure 4A). The associations between narcolepsy and obesity, any CM comorbidity, and any CV/CM/CR comorbidity were numerically stronger among the lower versus higher income groups, whereas the associations between narcolepsy and hypertension, hyperlipidemia, and any CV comorbidity were numerically stronger among higher versus lower income participants. For all outcomes assessed, the CIs for each income group overlapped, indicating nonsignificant differences between the groups (Figure 4B).

**Table 2:** Baseline comorbidities among participants with narcolepsy and matched participants without narcolepsy

	Narcolepsy cohort *n* = 694	Non-Narcolepsy cohort *n* = 2072
**Cardiovascular, *n* (%)**		
Angina pectoris	30 (4.3)	56 (2.7)
Atrial fibrillation	32 (4.6)	56 (2.7)
Heart failure	51 (7.4)	58 (2.8)
Myocardial infarction	28 (4.0)	43 (2.1)
Coronary arteriosclerosis	58 (8.4)	109 (5.3)
Hypertensive disorder	338 (48.7)	751 (36.3)
**Cardiometabolic, *n* (%)**		
Hyperlipidemia	232 (33.4)	543 (26.2)
Type 2 diabetes mellitus	124 (17.9)	242 (11.7)
Obesity	223 (32.1)	413 (19.9)
**Cardiorenal, *n* (%)**		
Chronic kidney disease	65 (9.4)	93 (4.5)
Hypertensive renal disease	41 (5.9)	59 (2.9)
**Psychiatric, *n* (%)**		
Attention deficit hyperactivity disorder	64 (9.2)	48 (2.3)
Generalized anxiety disorder	90 (13.0)	90 (4.3)
Depressive disorder	317 (45.7)	417 (20.1)
Bipolar disorder	78 (11.2)	67 (3.2)
Panic disorder	48 (6.9)	44 (2.1)
Post-traumatic stress disorder	57 (8.2)	64 (3.1)
Impaired cognition	66 (9.5)	41 (2.0)
**Neurological, *n* (%)**		
Restless leg syndrome	64 (9.2)	31 (1.5)
Epilepsy	45 (6.5)	46 (2.2)
Chronic pain syndrome	51 (7.4)	46 (2.2)
Musculoskeletal pain	347 (50.0)	660 (31.9)

### Incidence of CV/CM/CR conditions

The risk of developing hypertension, obesity, hyperlipidemia, and coronary arteriosclerosis was higher in the narcolepsy cohort compared with the non-narcolepsy cohort over the full follow-up time period, as well as at the 1-, 3-, and 5-year follow-up time points (Table S2). The highest incidence rates were at the 1 year follow-up in the narcolepsy cohort with 16.82 cases of hypertension, 11.51 cases of hyperlipidemia, and 13.58 cases of obesity, per 100 person years (Table S2).

After adjustment, the narcolepsy cohort had a higher risk of developing hypertension, hyperlipidemia, obesity, and coronary arteriosclerosis compared with the non-narcolepsy cohort over the full time period, as well as at 1, 3, and 5 years of follow-up (Table 3). In the full time period, the risk of developing hypertension was higher in the narcolepsy cohort compared with the non-narcolepsy cohort (HR: 2.00, 95 per cent CI: 1.51, 2.65). In addition, the risk of developing hyperlipidemia (HR: 1.96, 95 per cent CI: 1.52, 2.54), obesity (HR: 2.42, 95 per cent CI: 1.93, 3.05), and coronary arteriosclerosis (HR: 1.75, 95 per cent CI: 1.27, 2.42) was higher in the narcolepsy cohort compared with the non-narcolepsy cohort.

**Table 3:** Association between narcolepsy and the incidence of hypertension, hyperlipidemia, obesity, and coronary arteriosclerosis, over the total follow-up period and at 1, 3, and 5 years

	Narcolepsy cohort			Non-Narcolepsy cohort			HR (95% CI) ^*^
	Participants at risk, *n*	Events, *n*	Person-time in years	Participants at risk, *n*	Events, *n*	Person-time in years	
**Hypertension**							
Total	356	134	1988	1321	287	7166	2.00 (1.51, 2.65)
1 Year	327	55	327	1215	62	1215	3.84 (2.37, 6.22)
3 Year	262	80	786	953	143	2859	2.67 (1.89, 3.77)
5 Year	176	62	880	639	153	3195	2.25 (1.66, 3.06)
**Hyperlipidemia**							
Total	462	146	2464	1529	289	8090	1.96 (1.52, 2.54)
1 Year	417	48	417	1390	57	1390	3.63 (2.45, 5.37)
3 Year	327	85	981	1080	124	3240	2.89 (2.22, 3.78)
5 Year	212	74	1060	711	148	3555	2.42 (1.92, 3.05)
**Obesity**							
Total	471	132	2630	1659	228	9008	2.42 (1.93, 3.05)
1 Year	427	58	427	1504	51	1504	4.98 (3.32, 7.47)
3 Year	349	86	1047	1186	100	3558	3.17 (2.38, 4.23)
5 Year	236	72	1180	797	115	3985	2.75 (2.13, 3.55)
**Coronary arteriosclerosis**							
Total	636	65	3391	1963	110	10 275	1.75 (1.27, 2.42)
1 Year^†^	—	—	—	—	—	—	—
3 Year	449	21	1347	1363	43	4089	1.61 (1.00, 2.61)
5 Year	298	27	1490	897	44	4485	1.93 (1.31, 2.83)

^*^Adjusted for age at index, sex at birth (male, female, unknown), time in the EHR, race/ethnicity (non-Hispanic White, non-Hispanic Black, non-Hispanic Asian/Pacific Islander, other, unknown), income (<$50 k, ≥$50 k), education (college graduate, some college, high school/GED, unknown), employment status (employed, retired, out of work, not in workforce, unknown), disability status (yes, no, missing), and insurance type (commercial, Medicare, Medicaid, uninsured, other, prefer not to answer).

^†^Sample size too small for evaluation.

CI, confidence interval; EHR, electronic health record; GED, General Education Development; HR, hazard ratio.

### Healthcare resource utilization

Over both the baseline and follow-up periods, participants with narcolepsy were more likely to have had at least one inpatient stay or ED visit, compared with participants without narcolepsy; differences between the cohorts were smaller for outpatient visits but consistent with this trend (Table S3). Similarly, the narcolepsy cohort had higher unadjusted HCRU PPPM across inpatient, outpatient, and ED settings compared with the non-narcolepsy cohort (Table S3).

In adjusted models, the number of inpatient stays was significantly higher in the narcolepsy cohort compared with the non-narcolepsy cohort (incidence rate ratios [IRR]: 2.07, 95 per cent CI: 1.55, 2.78) during the baseline period (Table S4). The narcolepsy cohort also had a significantly higher number of outpatient (IRR: 1.51, 95 per cent CI: 1.35, 1.70) and ED (IRR, 1.70, 95 per cent CI: 1.19, 2.43) visits during the baseline period, compared with the non-narcolepsy cohort.

## Discussion

This study provides a comprehensive examination of the clinical, social, and economic burden of narcolepsy, highlighting unique features of vulnerable subgroups. By leveraging the *All of Us* Research Program, this study identified important patterns and disparities in HCRU, comorbid conditions, and socioeconomic status among participants with narcolepsy compared with participants without narcolepsy.

The narcolepsy cohort had a higher prevalence of CV, CM, CR, psychiatric, and neurological comorbidities at baseline, compared with the non-narcolepsy cohort. In adjusted models, composite outcome (CV/CM/CR) results were similarly higher in the narcolepsy cohort. In stratified analyses, the associations between narcolepsy and certain CM comorbidities at baseline (obesity and any CM comorbidity composite outcome) and between narcolepsy and any CV/CM/CR comorbidity, were numerically stronger in the lower versus higher income group. Even after accounting for other factors, narcolepsy was also associated with a higher incidence of hypertension, hyperlipidemia, obesity, and coronary arteriosclerosis across the total and 1-, 3-, and 5-year follow-up periods. HCRU was higher among those with narcolepsy both at baseline and in the follow-up period in both unadjusted and adjusted models. There is a growing body of evidence that SDoH, such as socioeconomic status, race/ethnicity, insurance coverage, and neighborhood deprivation, play a critical role in shaping both clinical and social outcomes in neurological disorders [20, 21]. One of the unique features of the current study is the additional in-depth SDoH information available for participants in *All of Us*. In addition to having a higher prevalence of psychiatric conditions and higher HCRU, the narcolepsy cohort more frequently reported seeing a mental health professional in the last year compared with the non-narcolepsy cohort. They were also more likely to report that they needed care and could not afford it or had to delay care for any reason. A larger proportion of the narcolepsy cohort had an annual income <$25 k and were unemployed, compared with the non-narcolepsy cohort. Moreover, participants with narcolepsy reported higher levels of loneliness and somewhat higher levels of everyday discrimination and medical discrimination than the non-narcolepsy cohort, in addition to lower social cohesion and social support.

The findings of this study are consistent with a broad literature examining the clinical, economic, and social burden of narcolepsy. A number of retrospective epidemiological studies have demonstrated that individuals with narcolepsy experience a wide array of medical comorbidities, compared with matched individuals without narcolepsy [3, 6–8, 22, 23]. These include an increased prevalence of CV diseases and outcomes, such as hypertension, and non-CV comorbidities associated with the risk of CV disease, such as comorbid sleep apnea, psychiatric disorders (e.g. depression and anxiety), metabolic disorders (e.g. diabetes), and obesity [3, 6–8, 22–24]. A review by Gudka in 2022 highlighted that the comorbidity burden of narcolepsy encompasses multiple organ systems, with mental and behavioral conditions, such as anxiety and mood disorders, being the most commonly identified comorbidities, followed by obesity and metabolic disorders, including diabetes and dyslipidemia [25]. In a community sample, Cohen *et al*. [3] found that having narcolepsy was associated with more than twice the likelihood of having hypertension, hyperlipidemia, or obesity. Using claims data, the BOND study showed the odds of stroke, myocardial infarction, cardiac arrest, heart failure, and coronary revascularization for 9312 individuals with narcolepsy were each higher (1.6–2.6 times) than the odds among those without narcolepsy [6]. Similarly, an interview-based study by Ohayon in 2013 found 32 per cent increased odds of hypertension, 51 per cent increased odds of hypercholesterolemia, and increased odds of heart disease, comparing adults with narcolepsy (*n* = 320) with a matched cohort of adults without narcolepsy (*n* = 1464) [23]. One study did not find a higher prevalence of hyperlipidemia or hypertension among individuals with narcolepsy compared with those without; however, that study used EHR data from a single referral center serving a clinically challenging population, and therefore, may not be representative of people either with or without narcolepsy in the United States [4]. The current study expands on prior work by demonstrating a higher prevalence of multiple CV and CM conditions in combined endpoints. These findings provide a broader perspective on the cumulative disease burden in narcolepsy, capturing increased risk across multiple organ systems rather than isolated conditions.

The body of literature examining the relationship between narcolepsy and the risk of developing CV/CM/CR outcomes is still emerging. The Cardiovascular Burden of Narcolepsy Disease (CV-BOND) study [5] found that narcolepsy was significantly associated with a higher risk of stroke and CV disease, including atrial fibrillation, heart failure, and major adverse CV events. Similarly, a study by Kaufmann *et al*. found that, compared with propensity-score–matched individuals without narcolepsy, individuals with narcolepsy had an increased risk of developing hypertension (HR: 1.40, 95 per cent CI: 1.34, 1.47), hyperlipidemia (HR: 1.41, 95 per cent CI: 1.35, 1.47), diabetes (HR: 1.50, 95 per cent CI: 1.38, 1.64), nonalcoholic fatty liver disease/nonalcoholic steatohepatitis (HR: 1.48, 95 per cent CI: 1.28, 1.73), CV disease composite (HR:1.61, 95 per cent CI: 1.35, 1.47), and major adverse CV events (HR: 1.69, 95 per cent CI: 1.43, 2.00) [9]. In addition, this elevated risk starts early in life (childhood and young adulthood) and persists throughout adulthood [9]. The same research group showed that individuals with narcolepsy had significantly higher risks of CV disease and major adverse cardiac events compared with those without narcolepsy, even after adjusting for stimulant medication use [26]. Furthermore, certain narcolepsy medications pose CV risks or contain elevated sodium, potentially compounding the substantial comorbidity burden observed in this population [27]. This is important to consider when planning a treatment strategy, as medication selection should balance treatment goals with the potential impact on CV health and comorbid conditions.

In addition to a higher prevalence and incidence of CV, CM, and CR comorbidities, the narcolepsy cohort also experienced greater HCRU compared with the non-narcolepsy cohort. Similarly, Flores *et al*. [2] found higher numbers of hospitalizations, ED visits, and outpatient visits in those with narcolepsy compared with matched individuals without narcolepsy, and Saad *et al*. [28] observed that individuals with narcolepsy experienced a high HCRU and medical cost burden; however, no direct comparison was made with controls.

While few studies have thoroughly investigated the relationship between SDoH and outcomes in narcolepsy, research has described the profound social burden that narcolepsy can impose. Jennum *et al*. found that individuals with narcolepsy were less likely to be married or cohabiting and had lower employment rates and income levels compared with matched controls, [29] highlighting the individual social and economic impact of narcolepsy. In a global survey of individuals with narcolepsy (NT1 and NT2), Flygare *et al*. noted that only 39 per cent of those with NT1 and 30 per cent of those with NT2 felt well-supported in adjusting to life with narcolepsy, and individuals who were older, unmarried, or did not know anyone else with their condition felt less supported [30]. Among young adults (ages 18–39) with narcolepsy who completed an online survey [31], almost all individuals said that living with narcolepsy made their social lives more challenging, and they also reported receiving more support from significant others compared with friends or family. These findings are consistent with the current study, in which the narcolepsy cohort experienced poorer outcomes across a range of SDoH factors, including social support, social cohesion, and loneliness.

Following the Centers for Medicare & Medicaid Services 2021 guidance encouraging healthcare systems to adopt value-based strategies aimed at reducing costs while improving patient outcomes, the US healthcare industry is beginning to shift from traditional fee-for-service payment models toward value-based models [32]. Many large insurers in the US have adopted real-world evidence and observational research strategies to better capture the diverse backgrounds, experiences, and outcomes of their patient populations. These efforts highlight the growing recognition of SDoH research across a broad range of disease areas, particularly those disproportionately affecting marginalized communities. The current study contributes to the growing acknowledgement of the role and impacts of SDoH on narcolepsy and clinical outcomes.

### Strengths and limitations

The strengths of this study include the relatively large sample size, the ability to investigate SDoH measures, and the combined survey and EHR data structure of *All of Us*. Owing to the larger sample size, more thorough investigations could be undertaken, including stratified and adjusted analyses to examine whether the associations of narcolepsy with outcomes varied by SDoH measures and to control for potential confounders. Additionally, the study design permitted the capture of both the prevalence and incidence of comorbidities in the baseline and follow-up periods, respectively, as well as the observation of HCRU in both the baseline and follow-up periods. Furthermore, the study design incorporated a similar time in the EHR for those with and without narcolepsy to allow for valid comparisons of prevalence between the cohorts.

However, several important limitations should be considered when interpreting the results of this study. The *All of Us* dataset inherently presents certain constraints typical of survey-based research, including potential response bias stemming from self-reported data. Specifically, participants completing voluntary surveys may systematically differ from those who do not respond, potentially introducing selection bias related to health literacy, engagement with healthcare services, and technology access. Furthermore, due to its recruitment approach, the *All of Us* cohort is not nationally representative. The demographic composition tends to skew older and more female, with inconsistent geographic representation across US states, which may limit the generalizability of our findings. However, it should be noted that the population examined was more racially and ethnically diverse than that of other observational studies in narcolepsy [4, 19, 31] and closer to the racial and ethnic composition of the United States. The data supporting our findings are less dominated by majority groups and may, therefore, be more reflective of population-level patterns. The cross-sectional nature of the survey responses presents additional limitations: critical variables, such as insurance coverage, employment status, and socioeconomic factors, may have changed since the time of data collection, potentially misclassifying certain exposures or covariates. Reliance on EHR data also introduced limitations. EHRs typically capture care provided within specific health systems and may fail to document healthcare utilization occurring out of network. Thus, HCRU, including specialist visits or emergency care received externally, may be underreported, potentially underestimating the true healthcare burden among individuals with narcolepsy. While the association may differ between narcolepsy subtypes (NT1 and NT2), subgroup analyses were not conducted due to small samples sizes and potential misclassification based on ICD diagnostic codes.

## Conclusions

The findings from this study not only reinforce existing knowledge but also uncovered novel insights with implications for risk management and policy. Specifically, the study highlights the need for timely and comprehensive intervention strategies to address whole-person health, taking into account the heightened prevalence and risk of CV, CM, CR, and psychiatric comorbidities, as well as potentially compromised SDoH in this vulnerable population. Additionally, identifying socioeconomic and healthcare access disparities can inform targeted public health initiatives aimed at improving care coordination and reducing barriers to essential treatments. Insights from this study support evidence-based policy recommendations that could influence insurance coverage, disability accommodation, and workforce policies to better support individuals with narcolepsy. Understanding whether earlier or specific interventions can mitigate clinical risks and disproportionate socioeconomic vulnerabilities is critical for improving long-term outcomes among those with narcolepsy.

## Supplementary Material

Markt-All_of_Us_MAN-Revision_2-SUPPLEMENTARY_INFORMATION-CLEAN_zpag088

## Data Availability

All relevant data are provided within the manuscript and supporting files.
