# Supplementary material for: Social determinants of health, clinical outcomes, and healthcare resource utilization in participants with and without narcolepsy in the All of Us research program
Source: Sleep Adv. 2026 Aug 5;7(3):zpag088. doi: 10.1093/sleepadvances/zpag088 (PMC13570643; doi:10.1093/sleepadvances/zpag088)
Supplement: Supplementary file 1 [file markt-all_of_us_man-revision_2-supplementary_information-clean_zpag088.docx]

**SUPPLEMENTARY MATERIAL**

**Social Determinants of Health, Clinical Outcomes, and Healthcare Resource Utilization in Participants With and Without Narcolepsy in the *All of Us* Research Program**

Sarah C. Markt^1^; Caroleen Drachenberg^1^; Anan Zhou^2^; Katarina Irwin^3^; Silky Beaty^1^; Jon Michael Stroh^3^; Martin Zagari^3^; Jessica K. Alexander^1^; Marisa Whalen^4^; Junko Saber^3^; Barbara Hutchinson^5^

^1^Jazz Pharmaceuticals, Palo Alto, CA, USA; ^2^Genesis Research Group, Hoboken, NJ, USA; ^3^Innopiphany, Irvine, CA, USA; ^4^Jazz Pharmaceuticals, Philadelphia, PA, USA; ^5^Chesapeake Cardiac Care, Bowie, MD, USA

**Corresponding Author**

Sarah C. Markt

Jazz Pharmaceuticals

3000 El Camino Real

2 Palo Alto Square

Suite 600

Palo Alto, CA 94306

[Sarah.Markt@jazzpharma.com](mailto:Sarah.Markt@jazzpharma.com)

### Supplemental Table 1. SNOMED CT Codes for Specific Comorbidities

| **Comorbidity** | **SNOMED CT Code** |
| --- | --- |
| **Cardiovascular** |  |
| Angina pectoris | 194828000 |
| Atrial fibrillation | 49436004 |
| Heart failure | 84114007 |
| Myocardial infarction | 22298006 |
| Cardiac arrest | 410429000 |
| Coronary arteriosclerosis | 53741008 |
| Hypertensive disorder | 38341003 |
| **Cardiometabolic** |  |
| Hyperlipidemia | 55822004 |
| Type 2 diabetes mellitus | 44054006 |
| Obesity | 414916001 |
| Metabolic encephalopathy | 50122000 |
| **Cardiorenal** |  |
| Chronic kidney disease | 709044004 |
| Hypertensive renal disease | 38481006 |
| **Psychiatric** |  |
| Attention deficit hyperactivity disorder | 406506008 |
| Generalized anxiety disorder | 21897009 |
| Depressive disorder | 35489007 |
| Bipolar disorder | 13746004 |
| Panic disorder | 371631005 |
| Seasonal affective disorder | 247803002 |
| Posttraumatic stress disorder | 47505003 |
| Impaired cognition | 386806002 |
| **Neurological** |  |
| Restless legs | 32914008 |
| Periodic limb movement disorder | 418763003 |
| Epilepsy | 84757009 |
| Chronic pain syndrome | 82423001 |
| Rapid eye movement sleep behavior disorder | 415238003 |
| Musculoskeletal pain | 279069000 |
| **Sleep (descriptive analysis only)** |  |
| Insomnia | 193462001 |
| Sleep apnea | 73430006 |

SNOMED CT, Standardized Nomenclature for Medicine–Clinical Terms.

### Supplemental Table 2. Unadjusted Incidence Rates of Select Comorbidities per 100 Person-Years

| **Outcome** | **Narcolepsy Cohort**  **IR (95% CI)** | **Non-Narcolepsy Cohort**  **IR (95% CI)** |
| --- | --- | --- |
| **Hypertension** |  |  |
| Total | 6.74 (5.91, 7.57) | 4.01 (3.16, 4.85) |
| 1 Year | 16.82 (14.86, 18.78) | 5.10 (3.14, 7.06) |
| 3 Years | 10.18 (9.05, 11.31) | 5.00 (3.87, 6.13) |
| 5 Years | 7.05 (6.17, 7.92) | 4.79 (3.91, 5.67) |
| **Obesity** |  |  |
| Total | 5.02 (4.19, 5.85) | 2.53 (1.69, 3.37) |
| 1 Year | 13.58 (11.62, 15.54) | 3.39 (1.43, 5.35) |
| 3 Years | 8.21 (7.08, 9.35) | 2.81 (1.68, 3.94) |
| 5 Years | 6.10 (5.23, 6.98) | 2.89 (2.01, 3.76) |
| **Hyperlipidemia** |  |  |
| Total | 5.93 (5.08, 6.77) | 3.57 (2.72, 4.42) |
| 1 Year | 11.51 (9.55, 13.47) | 4.10 (2.14, 6.06) |
| 3 Years | 8.67 (7.53, 9.80) | 3.83 (2.70, 4.96) |
| 5 Years | 6.98 (6.10, 7.86) | 4.16 (3.29, 5.04) |
| **Coronary arteriosclerosis** |  |  |
| Total | 1.92 (1.07, 2.77) | 1.07 (0.21, 1.93) |
| 1 Year^a^ | — | 1.25 (−0.71, 3.21) |
| 3 Years | 1.56 (0.43, 2.69) | 1.05 (−0.08, 2.18) |
| 5 Years | 1.81 (0.94, 2.69) | 0.98 (0.10, 1.86) |

^a^At 1 year, coronary arteriosclerosis events were <20 among participants with narcolepsy and thus blinded.

CI, confidence interval; IR, incidence rate.

### Supplemental Table 3. Unadjusted Healthcare Resource Utilization

|  | **Narcolepsy Cohort**  **n=694** | **Non-Narcolepsy Cohort**  **n=2072** |
| --- | --- | --- |
| **Baseline, n (%)** | 690 | 2012 |
| ≥1 Inpatient stay | 237 (34.3) | 501 (24.9) |
| ≥1 Outpatient visit | 655 (94.9) | 1827 (90.8) |
| ≥1 ED visit | 294 (42.6) | 654 (32.5) |
| **Baseline PPPM, mean (SD)** |  |  |
| Inpatient stay, days | 0.25 (0.55) | 0.14 (0.23) |
| Outpatient visits | 1.34 (1.84) | 0.77 (1.16) |
| ED visits | 0.21 (0.36) | 0.15 (0.30) |
| **Follow-up, n (%)** | 692 | 1856 |
| ≥1 Inpatient stay | 246 (35.6) | 481 (25.9) |
| ≥1 Outpatient visit | 671 (97.0) | 1798 (96.9) |
| ≥1 ED visit | 341 (49.3) | 673 (36.3) |
| **Follow-up PPPM, mean (SD)** |  |  |
| Inpatient stay, days | 0.30 (0.70) | 0.21 (0.70) |
| Outpatient visits | 2.18 (2.65) | 1.03 (1.42) |
| ED visits | 0.22 (0.32) | 0.15 (0.27) |

ED, emergency department; PPPM, per person per month; SD, standard deviation.

### Supplemental Table 4. Adjusted^a^ Healthcare Resource Utilization in the Baseline and Follow-up Periods

|  | **Baseline Period** | | **Follow-Up Period** | |
| --- | --- | --- | --- | --- |
|  | **IRR** | **95% CI** | **IRR** | **95% CI** |
| Outpatient | 1.51 | 1.35, 1.70 | 1.93 | 1.74, 2.15 |
| Inpatient | 2.07 | 1.55, 2.78 | 2.03 | 1.45, 2.83 |
| ED | 1.70 | 1.19, 2.43 | 2.05 | 1.64, 2.56 |

^a^Models adjusted for age at diagnosis, race/ethnicity, income, education, employment status, insurance, Charlson Comorbidity Index, Area Deprivation Index, disability status, regular place for healthcare advice, and delayed care.

CI, confidence interval; ED, emergency department; IRR, incidence rate ratio.
